# Supplementary material for: NGT1 Is Essential for N-Acetylglucosamine-Mediated Filamentous Growth Inhibition and HXK1 Functions as a Positive Regulator of Filamentous Growth in Candida tropicalis
Source: Int J Mol Sci. 2020 Jun 5;21(11):4036. doi: 10.3390/ijms21114036 (PMC7312872; doi:10.3390/ijms21114036)
Supplement: Supplementary file 1 [file ijms-21-04036-s001.pdf]

Supplementary Data

NGT1 is Essential for *N*-acetylglucosamine-mediated Filamentous Growth Inhibition and *HXK1* Functions as a Positive Regulator of Filamentous Growth in *Candida tropicalis*

Qiuyu Zhang, Li Xu, Sheng Yuan, Qinghua Zhou, Xuxia Wang, Lei Wang, Zhiming Hu, Yunjun Yan*

Key Laboratory of Molecular Biophysics, the Ministry of Education; College of Life Science and Technology, Huazhong University of Science and Technology, Wuhan 430074, P. R. China; qiuyuzhang2008@163.com (Q.Z.); xuli@mail.hust.edu.cn (L.X.) ; m201871755@hust.edu.cn (S.Y.) ; qinghuazhou1@126.com (Q.Z.); 2017506005@hust.edu.cn (X.W.) ; jiali20042005@126.com (L.W.) ; zhiming711@163.com (Z.H.)

* Correspondence: yanyunjun@hust.edu.cn; Tel.: +86-27-87792213

Table S1 Primers used in this study.

name	Sequence (5′ to 3′)	Purpose
NGT1-fwd	AGTTAAGCGGCCGCATGTCTTACCCAACTGAAG	*NGT1* overexpression
NGT1-rev	ACACTACTGCAGATTGTCTATGCTTGTCGATG	
NGT1-UP 5′	TGTATAGGGCCCAAGCTGTACCAACTAATGG	For *NGT1* knockout plasmid pSFS2A-NGT1KO
NGT1-UP 3′	TACTAACTCGAGATGCTGTTGTTGTTGTGATG	
NGT1-DOWN 5′	ATACTACCGCGGACTGTGTTGTAAGCAAATG	
NGT1-DOWN 3′	AACGTAGAGCTCCCATGATCAGTATTCAATG	
NGT1-L 5′	GTATTGGTGCATCTATTAGTG	Fusion PCR for NGT1 knockout
NGT1-L 3′	ACAAGGCAAGCTAAACAGATCTTTAGCAACGACTCTAGTAC	
NGT1-R 5′	ATCCAGTGTCGAAAACGAGCTAAATGTTGGGTGCTATTGTC	
NGT1-R 3′	TCGACTTTAGACTTGACAAC	
NGT1 5′ check	ATGTATAGTCGTGTGCGATG	*NGT1* deletion confirmation (first copy)
NGT13′ check	TTCAAGTGCAGCGAATAAAG	
NGT1 5′ ORF	TGAGAACTAGATCATTGAATTC	*NGT1* deletion confirmation (second copy)
NGT1 3′ ORF	AAATAAGTGGCACGGCAATC	
HXK1-fwd	AGTATTGCGGCCGCATGATTAAAACTACTATTGAAACTGC	*HXK1* overexpression
HXK1-rev	ATCATAGGTACCTCACTCCAACGTGTTGATTG	
HXK1-UP 5′	TCATATGGGCCCAGACAGCGATGATGAAGATG	For HXK1 knockout plasmid pSFS2A-HXK1KO
HXK1-UP 3′	AGTTATCTCGAGTGGAGTAGCAGTTTCAATAG	
HXK1-DOWN 5′	ATCATTCCGCGGTAGTTCAATCATTGGTGCTG	
HXK1-DOWN 3′	ACATATGAGCTCTGACAAGTTCTTTAACCACG	
HXK1-L 5′	TGATGGAAGTTAATGATGTC	Fusion PCR for *HXK1* knockout
HXK1-L 3′	ACAAGGCAAGCTAAACAGATCTGTAACTAGTGGTTTCCAATG	
HXK1-R 5′	ATCCAGTGTCGAAAACGAGCTTGACGATCACATGAAGTTG	
HXK1-R 3′	ACATAACCAATTGTAACGTC	
HXK1 5′ check	TTAACCATTGCCAAGTTGAG	*HXK1* deletion confirmation (first copy)
HXK13′ check	GTTGGCTCCATCTGATTTAC	
HXK1 5′ ORF	ATGATCATGGATGTGCGTTC	*HXK1* deletion

HXK1 3′ ORF	TCATAATCGTCAGTCTCGTG	confirmation (second copy)
DAC1-fwd	AGTATAGCGGCCGCATGTCTTATACTAGATTCACTAATTGC	*DAC1* overexpression
DAC1-rev	TTCAGACTGCAGAGCCAATAACTACATTGTCG	
DAC1-UP 5′	TAGTATGGGCCCTCTACTGTTGGCTTTAATAG	For *DAC1*
DAC1-UP 3′	AGTTATCTCGAGATGGATCATTAGGTCATCTG	knockout
DAC1-DOWN 5′	ATCATTCCGCGGTGTGCAAAAAGTCTACAAGC	plasmid pSFS2A-DAC1KO
DAC1-DOWN 3′	ATTACAACAAGAGAGCTCAG	
DAC1-L 5′	TATAAACTCCACCATGTGTC	Fusion PCR for
DAC1-L 3′	ACAAGGCAAGCTAAACAGATCTAGTCACAGTAGGACAAGTAG	*DAC1* knockout
DAC1-R 5′	ATCCAGTGTCGAAAACGAGCTTGTACAATGCCATGCCAC	
DAC1-R 3′	TAGCCTTGTTTGTCCAATAC	
DAC1 5′ check	AGCATGTCTTGGAACTTTAG	*DAC1* deletion
DAC13′ check	ATCATTAGCCGCTCCATTG	confirmation (first copy)
DAC1 5′ ORF	CGAATTTCCCAGAGGTTTAC	*DAC1* deletion
DAC1 3′ ORF	TTCAATAGCTTGAACACCTG	confirmation (second copy)
NAG1-fwd	AGTTAAGCGGCCGCATGAGACAAGCTACTTTTTCCAG	*NAG1* overexpression
NAG1-rev	TTCAGACTGCAGTAGTTCAATCATTGGTGCTG	
NAG1-UP 5′	TAGTATGGGCCCAGAATCTCCTAAACTCAGTG	For *NAG1*
NAG1-UP 3′	AGTTATCTCGAGTGTATAGCTAAAGTGTCATGG	knockout plasmid
NAG1-DOWN 5′	ATCATACCGCGGTACTGGTGTTACTCACATTG	pSFS2A-NAG1KO
NAG1-DOWN 3′	AACGTAGAGCTCTGTTGATGCTAGATTCAAAC	
NAG1-L 5′	TCCGAATTGTAATGATGATG	Fusion PCR for
NAG1-L 3′	ACAAGGCAAGCTAAACAGATCTAGTGGCCAATCCATTCAAG	*NAG1* knockout
NAG1-R 5′	ATCCAGTGTCGAAAACGAGCTTTATTTTTGGGTGGCTTGG	
NAG1-R 3′	CTCATAGTCTACTTTGTCTTG	
NAG1 5′ check	CAAGTATTTTGTCATGGCAT	*NAG1* deletion
NAG1 3′ check	ATTTGCCACCAAGTATGATG	confirmation (first copy)
NAG1 5′ ORF	TGTGCTGAATATGAAGCTAAG	*NAG1* deletion
NAG1 3′ ORF	TTTAGATTTCAAGCCAGCAG	confirmation

		(second copy)
Hph-F	AGATCTGTTTAGCTTGCCTTG	Amplification cahph marker for fusion PCR
Hph-R	AGCTCGTTTTCGACACTGG	
NGT-F	TTGGACTTTGGAGAACTTTG	For q-RT-PCR
NGT1-R	ACATTTGAGCTAACCAGTAC	
HXK1-F	AACCACTAGTTACAACAGTG	
HXK1-R	ACATCCATGATCATTCCATG	
DAC1-F	TTCTACAAAGATGCCATGAC	
DAC1-R	AGTCTGGCCATCCAATCTAG	
NAG1-F	TTGGCTCCATCTGATTTAC	
NAG1-R	TCTTAGCTTCATATTCAGCAC	
ACT1-F	TTGTACTCTTCTGGTAGAAC	
ACT1-R	AAGTGTAACCACGTTCAGAC	
